# Circular RNA hsa_circ_0007990 as a blood biomarker for unruptured intracranial aneurysm with aneurysm wall enhancement

**DOI:** 10.3389/fimmu.2022.1061592

**Published:** 2022-11-17

**Authors:** Xiao-Bing Wu, You-Tao Wu, Xin-Xing Guo, Chun Xiang, Pei-Sheng Chen, Wang Qin, Zhong-Song Shi

**Affiliations:** ^1^ Department of Neurosurgery, Sun Yat-sen Memorial Hospital, Sun Yat-sen University, Guangzhou, China; ^2^ RNA Biomedical Institute, Sun Yat-sen Memorial Hospital, Sun Yat-sen University, Guangzhou, China; ^3^ Guangdong Province Key Laboratory of Brain Function and Disease, Sun Yat-sen University, Guangzhou, China

**Keywords:** aneurysm wall enhancement, blood marker, circular RNA, intracranial aneurysm, vessel wall imaging, inflammation

## Abstract

**Background:**

Circular RNAs (circRNAs) may involve the formation and rupture of intracranial aneurysms (IA). Inflammation plays a vital role in the development and progression of IA, which can be reflected by aneurysm wall enhancement (AWE) on high-resolution vessel wall magnetic resonance imaging (HR-VWI). This study aims to evaluate the role of circRNAs as the blood inflammatory biomarker for unruptured IA (UIA) patients with AWE on HR-VWI.

**Methods:**

We analyzed the circRNA expression profiles in the peripheral blood samples among subjects from saccular UIA with AWE, UIA without AWE, and healthy controls by the circRNA microarray. The differential expression of hsa_circ_0007990 was assessed. We constructed the hsa_circ_0007990-microRNA-mRNA network and the regulatory axis of hub genes associated with the AWE in UIA.

**Results:**

Eighteen patients harboring saccular UIAs with HR VWI and five healthy controls were included. We found 412 differentially expressed circRNAs between UIA patients and healthy controls by circRNA microarray. Two hundred thirty-one circRNAs were significantly differentially expressed in UIA patients with AWE compared with those without AWE. Twelve upregulated circRNAs were associated with AWE of UIA, including hsa_circ_0007990, hsa_circ_0114507, hsa_circ_0020460, hsa_circ_0053944, hsa_circ_0000758, hsa_circ_0000034, hsa_circ_0009127, hsa_circ_0052793, hsa_circ_0000301 and hsa_circ_0000729. The expression of hsa_circ_0007990 was increased gradually in the healthy control, UIA without AWE, and UIA with AWE confirmed by RT-PCR (P<0.001). We predicted 4 RNA binding proteins (Ago2, DGCR8, EIF4A3, PTB) and period circadian regulator 1 as an encoding protein with hsa_circ_0007990. The hsa_circ_0007990-microRNA-mRNA network containing five microRNAs (miR-4717-5p, miR-1275, miR-150-3p, miR-18a-5p, miR-18b-5p), and 97 mRNAs was constructed. The five hub genes (hypoxia-inducible factor 1 subunit alpha, estrogen receptor 1, forkhead box O1, insulin-like growth factor 1, CREB binding protein) were involved in the inflammatory response.

**Conclusion:**

Differentially expressed blood circRNAs associated with AWE on HR-VWI may be the novel inflammatory biomarkers for assessing UIA patients. The mechanism of hsa_circRNA_0007990 for UIA progression needs to investigate further.

## Introduction

Unruptured intracranial aneurysms (UIA) cause subarachnoid hemorrhage once bleeding, resulting in severe poor clinical outcomes. The risk of rupture in UIA significantly decreases after receiving endovascular treatment or surgical clipping in cases of aneurysm growth (1, 2). Inflammation plays a vital role in the development and progression of intracranial aneurysms (IA), which can be reflected by aneurysm wall enhancement (AWE) on high-resolution vessel wall magnetic resonance imaging (HR-VWI) (3–8). Recent studies suggest the association of inflammatory blood biomarkers in the aneurysm sac and peripheral blood with AWE on HR-VWI in patients with UIA (9–13).

Recent evidence shows that circular RNAs (circRNAs), one type of non-coding RNA, involve the formation and rupture of IA (14–19). CircRNAs regulate vascular smooth muscle cells phenotype involving the pathological process of IA (14–16). Differentially expressed circRNAs in the peripheral blood are associated with the aneurysm rupture status in patients with IA and may be used as potential clinical diagnostic biomarkers (20–22). However, the association of circRNAs in the peripheral blood with AWE on HR-VWI for IA remains unclear. In this study, we aim to evaluate the role of circRNAs as the blood inflammatory biomarker for UIA patients with AWE on HR-VWI.

## Materials and methods

### Patients selection

The institutional review board approved this study at Sun Yat-sen Memorial Hospital, Sun Yat-sen University. All participants signed their written informed consent to participate in this study. Patients with UIA for initial endovascular and surgical treatment from our HR-VWI aneurysm database were selected for this study between March 2018 and March 2021. Patients were older than 18 years old. Saccular UIA was confirmed by digital subtraction angiography and can be identified on MR angiography. Patients had images of HR-VWI without artifacts. We excluded patients with aneurysms in the extracranial or cavernous sinus of the internal carotid artery and with a size less than 3mm. Healthy adults were recruited for this study and had brain MR angiography examinations to exclude the presence of UIA and other intracranial lesions. All participants had no history of pneumonia, heart disease, cancer, autoimmune disease, hematological disease, chronic liver disease, kidney disease, or recent brain surgery. We recorded the data on medical history, laboratory examination, and clinical and radiological characteristics.

### HR-VWI study

Patients with UIA underwent brain MR for HR-VWI on a 3.0T MRI scanner. We divided the pattern of AWE into none, focal, and circumferential AWE according to the precontrast and postcontrast HR-VWI T1-weighted sequences described in our previous study. The aneurysm-to-pituitary stalk contrast ratio (CRstalk) was calculated using the maximal signal intensity value of the aneurysm wall (SIwall) and the pituitary stalk (SIstalk) on the 3D postcontrast HR-VWI T1-weighted sequence as CRstalk = SIwall/SIstalk. CRstalk was used as an optimal quantitative analysis of AWE in UIA (8, 12).

### Peripheral blood sample and RNA extraction

Before any antiplatelet drug administration or treatment, 2 ml peripheral venous blood was collected in the early morning after overnight fasting from all participants. We extracted total RNA from each peripheral whole blood sample using TRI Reagent BD as described in the manufacturer’s brochure (Molecular Research Center, Cincinnati, OH, United States) (23). RNA quality was assessed by the NanoDrop ND-1000 Spectrophotometer (Thermo Fisher Scientific, Waltham, MA, United States), and RNA with an OD260/OD280 ratio between 1.8 and 2.1 was considered good quality. Agarose gel electrophoresis was used to assess RNA integrity.

### RNA labeling and hybridization

The RNase R (Epicentre, Inc.) was used to remove linear RNAs to enrich the circular RNAs. Circular RNAs were amplified, transcribed into cRNA, and purified using an RNeasy Mini Kit (Qiagen) according to the manufacturer’s protocols. The purified cRNA was labeled using Quick Amp Labeling Kit (Agilent) at 40°C for 2 hours. The NanoDrop ND-1000 was used to determine the yield and specific activity of the labeled cRNAs. Then, cDNA samples were hybridized onto the Human circRNA Arrays (V2.0, Arraystar Inc). Hybridization was performed at 65°C for 17 hours in an Agilent Hybridization Oven. Hybridized arrays were washed, fixed, and scanned with a G2505C Agilent Microarray Scanner.

### circRNA microarray analysis

We obtained the original data by Agilent Feature Extraction software (version 11.0.1.1). The data were normalized and processed using the R software limma package. The expression level of circRNA was analyzed and compared among groups. False discovery rate (FDR) was calculated to account for multiple testing using R (version 3.6.7; R Foundation for Statistical Computing) (24). The difference in circRNAs expression was considered significant with Fold change (FC) ≥ 1.3 and an FDR-adjusted *P* value < 0.05.

### Quantitative real-time PCR

qRT-PCR was utilized to assess the significantly differentially expressed circRNAs screened from microarray analysis. Total RNA was extracted and used as a template for reverse transcription to first-strand cDNA with SYBR Premix Ex Taq II (Tli RNaseH Plus; TaKaRa, Osaka, Japan). qRT-PCR with a total 10μl reaction volume was performed on the Quanstudio DX (ABI) machine using GAPDH as an endogenous reference for normalization (25). The primer sequences for circRNAs are listed in **Supplementary Table 1**. Each sample was tested three times. The 2−ΔΔCt method was applied to obtain the relative circRNA expression.

### GO and KEGG pathway analysis

We used the circBank database to search microRNAs having a binding site with our target circRNA. Then, we predicted the simultaneous target mRNAs of the circRNA-targeted microRNAs by both TargetScan and miRDB databases. We performed the GO enrichment analysis and Kyoto Encyclopedia of Genes and Genomes (KEGG) pathway analysis to reveal the biological function of target genes predicted by the circRNA-binding microRNAs using the David (https://david.ncifcrf.gov). The GO enrichment analysis included the biological process, cellular component, and molecular function. Furthermore, the RNA binding proteins with target circRNA were searched by the CircInteractome database. The open reading frame of target circRNA and their potential encoding proteins were also analyzed using the online database.

### Construction circRNA-microRNA-mRNA network with hub gene prediction

We identified the crucial circRNA and its targeted microRNAs and genes to construct the circRNA-microRNA-mRNA network using Cytoscape software (version 3.9.1) for visualization. Then, predicted genes from the top ten enriched KEGG pathways from the target circRNA-microRNAs were selected to construct the protein-protein interaction network by the Search Tool for the Retrieval of Interacting Genes (STRING, version 11.5; https://cn.string-db.org/cgi/input). After obtaining gene interaction for those target genes, the top ten hub genes were defined from the calculation by the maximal clique centrality algorithm in the CytoHubba plugin. Finally, we obtained the circRNA-microRNA-mRNA network containing ten key regulatory axes using the Cytoscape.

### Statistical analysis

Continuous and categorical variables were analyzed by Student t-test, Mann-Whitney U test, Fisher’s exact, or chi-square test. A P-value less than 0.05 was considered significant for the results. Statistical analyses were performed using SPSS 22.0 software.

## Results

### CircRNA expression profiles in peripheral blood from UIA

**Figure 1:**
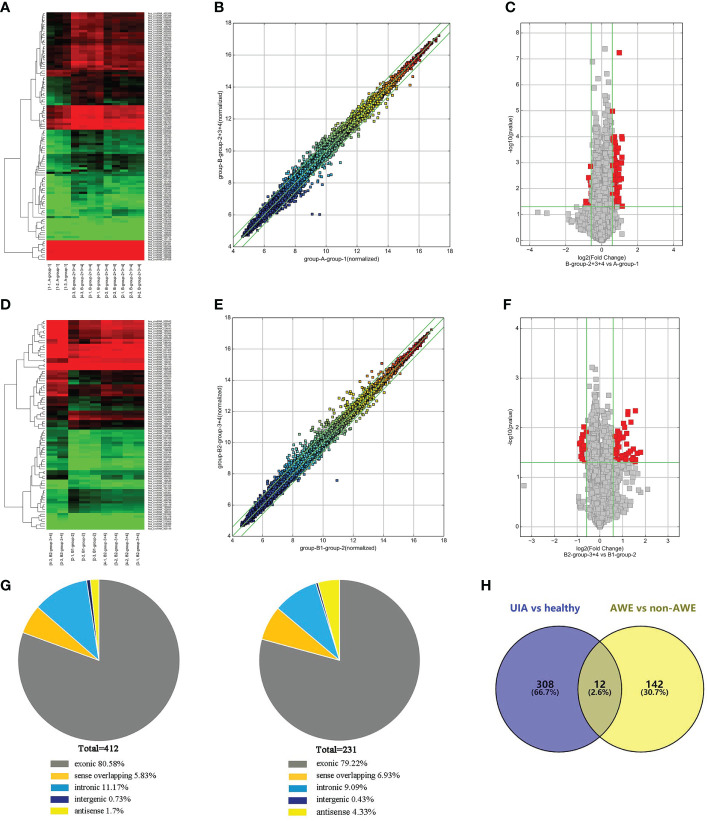
Differentially expressed circRNAs in peripheral blood identified by circRNAs microarray. **(A)** The hierarchical clustering of differentially expressed circRNAs between the unruptured intracranial aneurysm (UIA) and healthy control groups. Group A represented healthy control; group B represented the UIA group. **(B)** The scatterplot is used to assess the circRNA expression variation between the UIA and healthy control group of samples. The circRNAs above the top green line and below the green bottom line indicate more than a 1.3-fold change of circRNAs between the two compared samples. **(C)** Volcano Plots are used to visualize differential expression between the UIA and healthy control groups. The red point in the plot represents the differentially expressed circRNAs with statistical significance. **(D)** The hierarchical clustering of differentially expressed circRNAs between the UIA with aneurysm wall enhancement (AWE) and UIA without AWE. Group B1 represented UIA with AWE of samples; group B2 represented UIA without AWE of samples. **(E)** The scatterplot is used for assessing the circRNA expression variation between the UIA with AWE and UIA without AWE. The circRNAs above the top green line and below the green bottom line indicate more than a 1.3-fold change of circRNAs between the two compared samples. **(F)** Volcano Plots are used for visualizing differential expression between the UIA with AWE and UIA without AWE. The red point in the plot represents the differentially expressed circRNAs with statistical significance. **(G)** The genomic origin of the differentially expressed circRNAs among three groups of samples (UIA with AWE, UIA without AWE, and healthy controls). **(H)** A total of 12 significantly differentially expressed peripheral blood circRNAs among the three compared groups (UIA with AWE, UIA without AWE, and healthy controls) were identified by circRNA microarray.

We identified twenty-three subjects in this study, including 18 patients with UIAs and five healthy normal controls. The characteristics of all participants are shown in **Table 1**. We analyzed the circRNA expression profile of peripheral blood in six samples from UIA with AWE group, three from UIA without AWE, and three from normal controls by circRNA microarray. A total of 13 615 circRNAs were detected in the three groups using the Arraystar human circRNA Microarray V2.0. The differentially expressed peripheral blood circRNAs were generated and assessed by hierarchical clustering, scatter plots, and volcano plots between the UIA group and healthy control group (**Figure 1A-C**) and between UIA with AWE and UIA without AWE (**Figure 1D-F**).

**Table 1:** Clinical characteristics of participants with unruptured intracranial aneurysm and healthy control.

	UIA patients with AWE (n = 12)	UIA patients with non-AWE (n = 6)	Healthy control (n = 5)	P Value
Age (yr)	53.0 ± 13.9	45.2 ± 10.3	24.8 ± 2.5	0.001
Female	8 (66.7%)	2 (33.3%)	0	0.035
Hypertension	7 (58.3%)	2 (33.3%)	0	0.076
Smoking	2 (16.7%)	2 (33.3%)	0	0.379
Size	6.15 (3.8-16.2)	4.15 (3.2-6.5)	N/A	0.025
Location				0.083
Anterior circulation	11 (91.7%)	14 (77.8%)	N/A	
Posterior circulation	1 (8.3%)	3 (50%)	N/A	
Irregular shape	7 (58.3%)	2 (33.3%)	N/A	0.620
CRstalk	0.48 (0.32-0.75)	0.23 (0.07-0.26)	N/A	0.001
hsa_circ_0007990	4.19 ± 1.48	1.78 ± 0.51	1.00 ± 0.19	< 0.001

AWE, aneurysm wall enhancement; CRstalk, aneurysm-to-pituitary stalk contrast ratio; UIA, unruptured intracranial aneurysm. N/A, Not available.

We found 412 circRNAs significantly differentially expressed in the UIA group compared with the healthy control group (fold change ≥1.3, p-value <0.05). Among the differentially expressed circRNAs, 320 were upregulated while 92 were downregulated. We identified 231 circRNAs significantly differentially expressed in the UIA with AWE compared with the UIA without AWE. These included 154 upregulated circRNAs and 77 downregulated circRNAs. Differentially expressed circRNAs were classified into exonic, intronic, antisense, sense overlapping, and intergenic. The genomic origin of the differentially expressed circRNAs among three groups is shown in **Figure 1G**. Among the 412 differentially expressed circRNAs between the UIA and healthy control groups, 332 (80.58%) circRNAs are from exons. Among the 231 differentially expressed circRNAs between UIA with AWE and UIA without AWE, 183 (79.22%) circRNAs are from exons.

### Differentially expressed CircRNAs in UIA with AWE

We found a total of 12 significantly differentially expressed peripheral blood circRNAs among the three compared groups identified by circRNA microarray (**Figure 1H**). Among these 12 upregulated circRNAs in UIA with AWE, 10 (83.33%) have been identified in circBase, including hsa_circ_0007990, hsa_circ_0114507, hsa_circ_0020460, hsa_circ_0053944, hsa_circ_0000758, hsa_circ_0000034, hsa_circ_0009127, hsa_circ_0052793, hsa_circ_0000301, hsa_circ_0000729. In addition, 9 (75%) of 12 differentially expressed circRNAs consist of exons (**Table 2**).

**Table 2:** **A** total of 12 significantly differentially expressed peripheral blood circRNAs associated with aneurysm wall enhancement in UIA.

circRNA ID	circbase ID	Position	Gene	Chr	LogFC	P-value	Type	Regulation
hsa_circRNA_102062	hsa_circ_0007990	chr17: 37840849-37842272 strand: -	PGAP3	17	1.5440902	0.046706765	exonic	up
hsa_circRNA_400285	hsa_circ_0114507	chr1: 93580244-93581175 strand: +	MTF2	1	1.4553306	0.029050504	exonic	up
hsa_circRNA_100724	hsa_circ_0020460	chr10: 128850944-128860040 strand: +	DOCK1	10	1.4365029	0.01294899	exonic	up
hsa_circRNA_053944	hsa_circ_0053944	chr2: 33808728-33810511 strand: -	FAM98A	2	1.4354857	0.047122417	exonic	up
hsa_circRNA_000758	hsa_circ_0000758	chr17: 34151081-34165557 strand: +	TAF15	17	1.3864204	0.023292637	exonic	up
hsa_circRNA_100104	hsa_circ_0000034	chr1: 26772806-26774151 strand: +	DHDDS	1	1.3732708	0.04457919	exonic	up
hsa_circRNA_009127	hsa_circ_0009127	chr9: 36594624-36607670 strand: +	MELK	9	1.3232472	0.044595488	exonic	up
hsa_circRNA_102632	hsa_circ_0052793	chr2: 15735268-15735677 strand: +	DDX1	2	1.3094958	0.031947264	exonic	up
hsa_circRNA_001459	hsa_circ_0000301	chr11: 47379617-47379952 strand: -	SPI1	11	1.7396791	0.043873347	intronic	up
hsa_circRNA_001389	hsa_circ_0000729	chr16: 90098243-90098338 strand: +	GAS8	16	1.5706839	0.034928521	intronic	up
hsa_circRNA_404695	N/A	chr1: 244640842-244682000 strand: +	C1orf101	1	1.5649027	0.044375298	exonic	up
hsa_circRNA_406908	N/A	chr7: 7916911-7923977 strand: +	RPA3-AS1	7	1.3492812	0.043894901	intronic	up

N/A, Not available.

### Upregulation of hsa_circ_0007990 in peripheral blood of UIA with AWE

**Figure 2:**
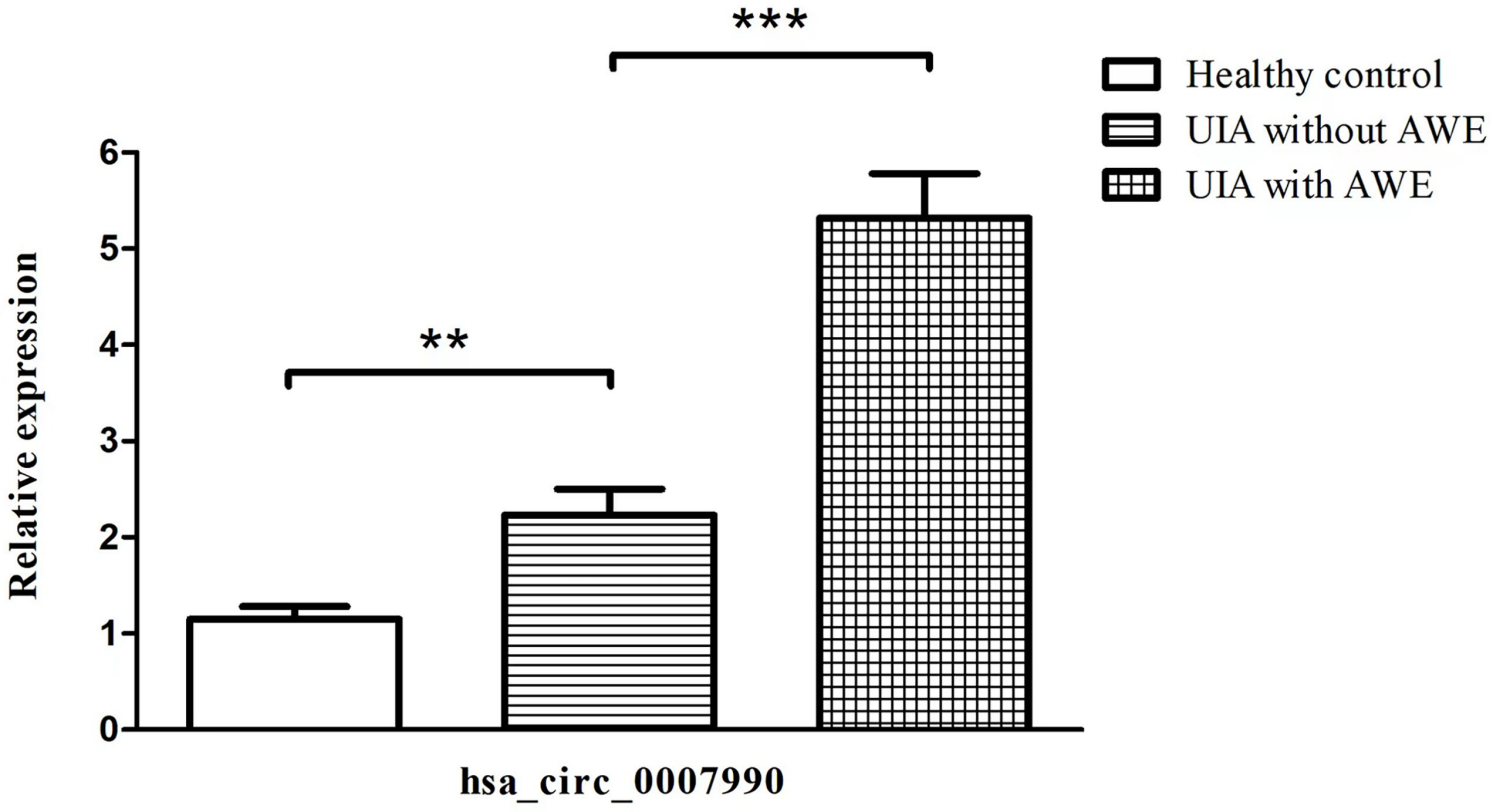
Upregulated expression of hsa_circ_0007990 associated with aneurysm wall enhancement confirmed by RT-PCR. The data are the mean ± SD, **p < 0.01, ***p < 0.001.

We selected hsa_circ_0007990 to compare the expression levels among the three groups by qRT-PCR because hsa_circ_0007990 has the most remarkable fold change among the differentially expressed exonic circRNA associated with UIA with AWE. The expression of peripheral blood hsa_circ_0007990 was significantly increased in turn among the group of healthy control, UIA without AWE, and UIA with AWE (*P*<0.001; **Figure 2**). The level of hsa_circ_0007990 was higher in the UIA group than in the healthy control group (3.38 vs. 1.00, *P*=0.005). The level of hsa_circ_0007990 was significantly higher in the UIA with AWE than those without AWE (4.19 vs. 1.78, *P*=0.001).

### Characteristics and functions of hsa_circ_0007990

**Figure 3:**
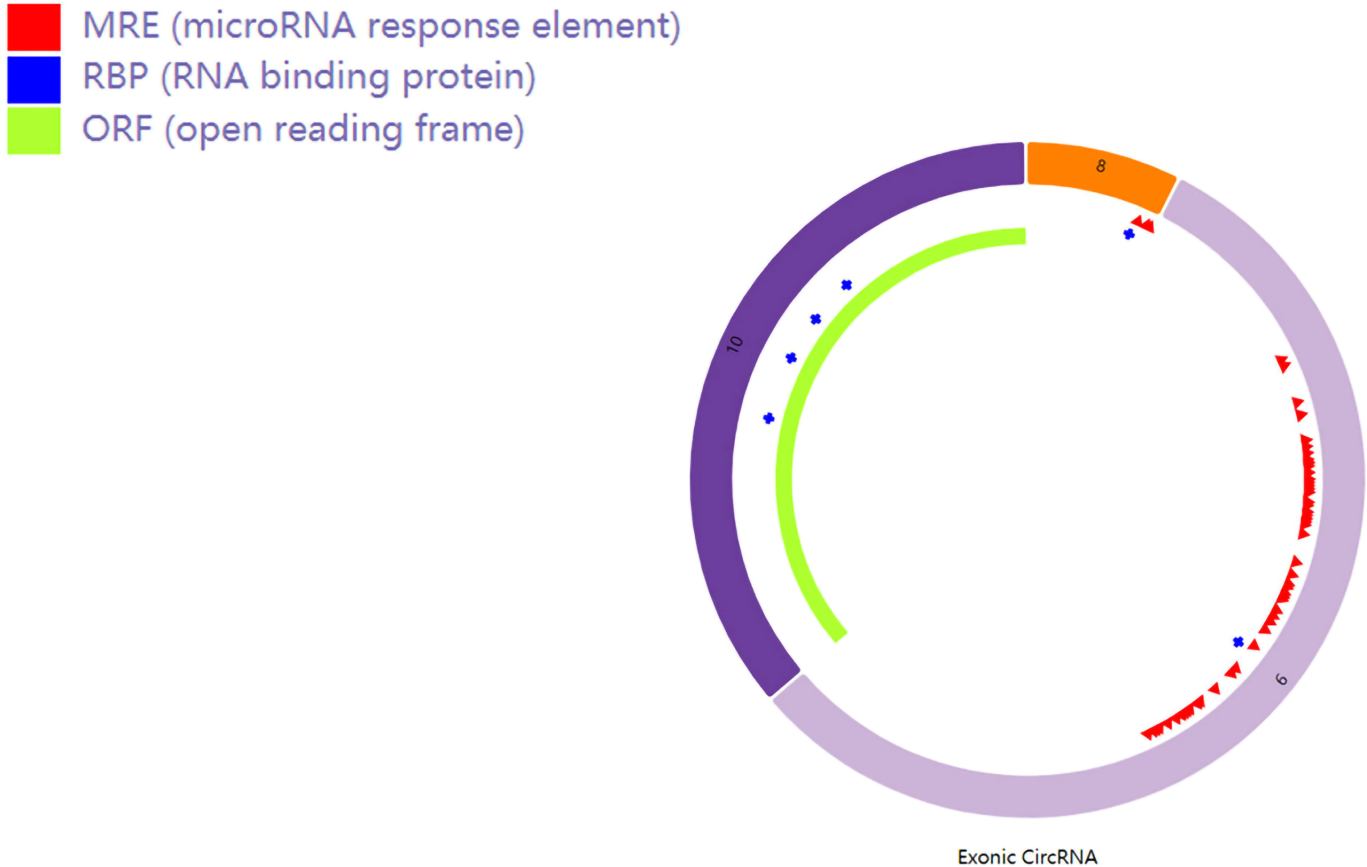
Schematic diagram of hsa_circ_0007990. The red bar represents the microRNA binding sites, the blue bar represents proteins binding with hsa_circRNA_0007990, and the light green bar represents the open reading frame.

**Figure 4:**
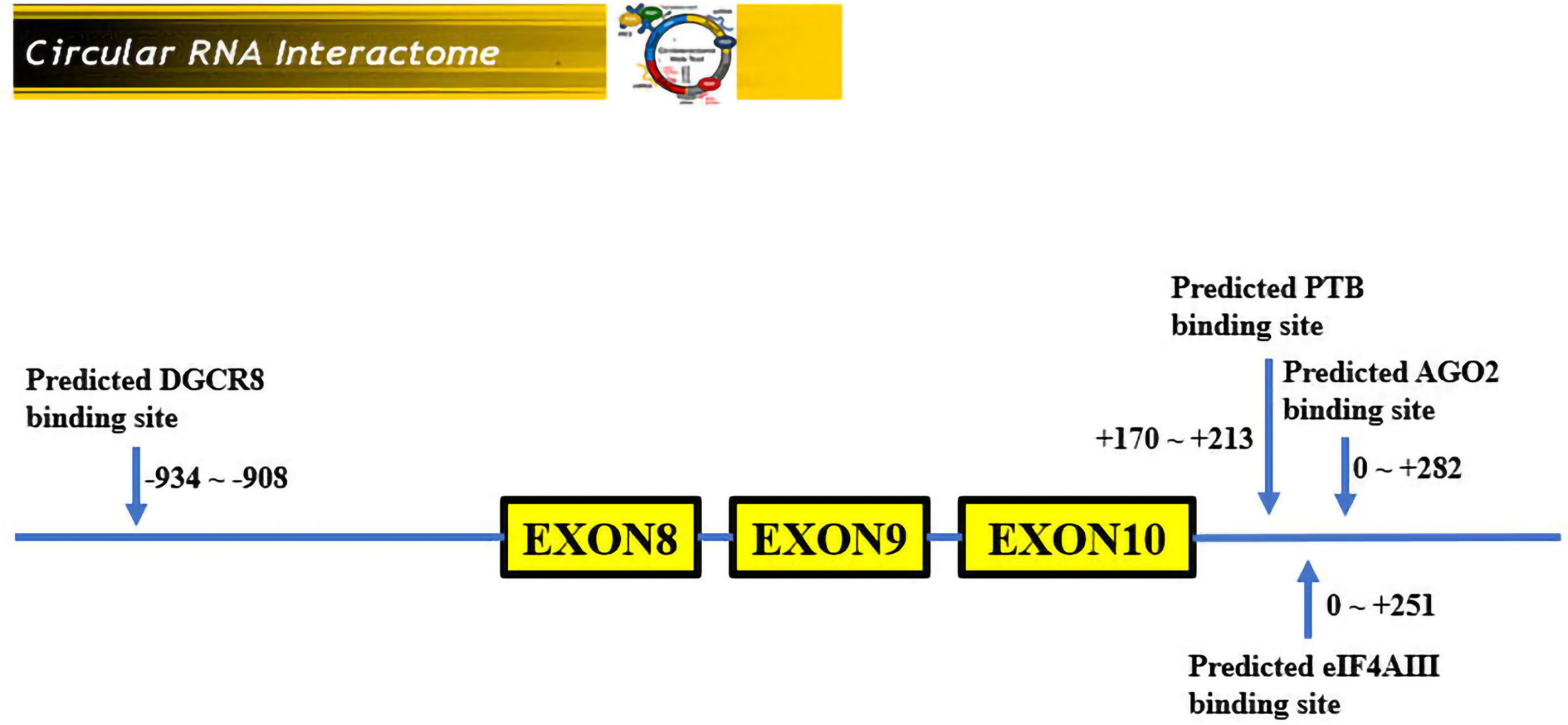
RNA binding proteins with hsa_circ_0007990. According to the CircInteractome database, the 3′start flanking intron upstream of hsa_circRNA_0007990 has a potential binding region with DGCR8 protein, and the 3′start flanking intron downstream of hsa_circRNA_0007990 has a potential binding region with Ago2, EIF4A3, and PTB protein.

Hsa_circRNA_0007990 is derived from exon 8, exon 9, and exon 10 of the PGAP3 gene (NM_033419) located on homo sapiens chromosome 17 with a sequence length of 251 bp, and its circBank ID is hsa_circPGAP3_003, which contains the open reading frame (ORF), microRNA binding sites, and the ability of RNA binding protein (**Figure 3**). One function of circRNA is as microRNA sponges to control gene transcription by binding with microRNAs. A total of 29 microRNAs having at least two binding sites with hsa_circRNA_0007990 were predicted by the circBank database (**Supplementary Table 2**). The top ten predicted microRNAs are as follows, including miR-4717-5p, miR-1275, miR-150-3p, miR-18a-5p, miR-18b-5p, miR-3914, miR-3934-5p, miR-4254, miR-433-5p. Furthermore, four RNA binding proteins with hsa_circRNA_0007990 (argonaute-2 [Ago2], DiGeorge syndrome critical region 8 [DGCR8], eukaryotic initiation factor 4A-III [EIF4A3], polypyrimidine tract binding [PTB] protein) were found from the CircInteractome database (**Figure 4**).

We had the nucleotide sequence of hsa_circRNA_0007990 from the CircBase database, and then four ORFs were detected by the online database ORFfinder (https://www.ncbi.nlm.nih.gov/orffinder). Among the four ORFs (**Table 3**), only the ORF2 has a function of encoding protein named as the period circadian regulator 1 (PER1) superfamily searched from the online database of Conserved Domains (https://www.ncbi.nlm.nih.gov/Structure/cdd/wrpsb.cgi).

**Table 3:** Open reading frames detected from hsa_circ_0007990.

Label	Strand	Frame	Start	Stop	Length (nt|aa)	Nucleotide sequence
ORF1	+	1	28	180	153|50	MSVCGSPLGSTSRKVTKCLSSMASGPSPGSCSFKSRHRPWPRFSMAWPAW
ORF2	+	3	36	>251	216|71	MWVTVGLYLQEGHKVPQFHGKWPFSRFLFFQEPASAVASFLNGLASLVML
ORF3	–	1	128	>3	126|41	CRYRTFVPASSPMYHTCVAFAW
ORF4	–	3	138	43	96|31	MKEQEPGEGPLAMELRHFVTFLEVEPNGDPHTLILTVVPTGP

### Gene oncology and KEGG pathway analysis of hsa_circ_0007990

**Figure 5:**
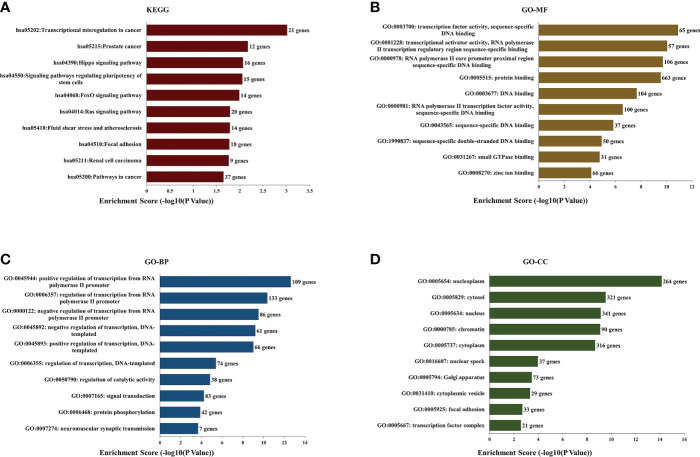
Kyoto Encyclopedia of Genes and Genomes (KEGG) pathway and Gene Ontology (GO) functional enrichment analysis of the predicted target genes from has_circRNA_0007990. **(A)** KEGG. **(B)** GO-molecular functions. **(C)** GO-biological processes. **(D)** GO-cell components.

We selected the top five predicted microRNAs binding on hsa_circRNA_0007990 through the CircBank database and searched the target mRNAs of these five microRNAs by both TargetScan and miRDB databases simultaneously (**Supplementary Table 3**). Then, we performed the GO and KEGG enrichment analysis to analyze the biological function of 961 target genes predicted by hsa_circRNA_0007990. The top ten enriched pathways from KEGG pathways analysis were found as follows: transcriptional misregulation in cancer, prostate cancer, Hippo signaling pathway, signaling pathways regulating pluripotency of stem cells, FoxO signaling pathway, Ras signaling pathway, fluid shear stress and atherosclerosis, focal adhesion, renal cell carcinoma, pathways in cancer (**Figure 5A**). The top ten enriched terms of biological process, cellular component, and molecular function of GO are shown in **Figure 5B-D**.

### CircRNA–microRNA–mRNA network construction

**Figure 6:**
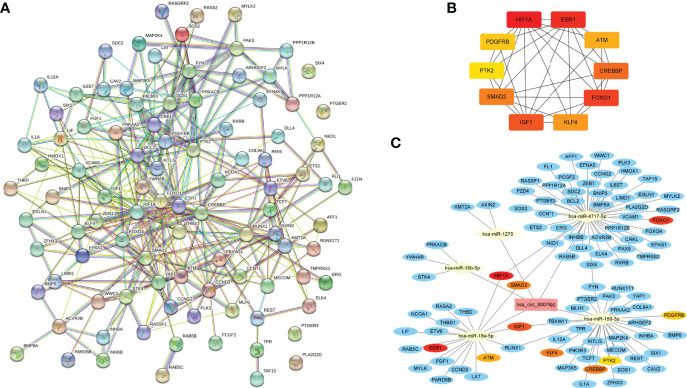
The circRNA-miRNA-mRNA regulatory network of hsa_circRNA_0007990. **(A)** Protein-protein interaction network obtained on STRING website. **(B)** Relationship network diagram of hub genes from protein-protein interaction network. **(C)** The reconstructed circRNA-microRNA-hub genes network.

Ninety-seven targeted genes from the top ten enriched KEGG pathways were selected to construct the protein-protein interaction network by the STRING online tool (**Figure 6A** and **Supplementary Table 4**). Then the top ten hub genes were calculated by the MCC method in the CytoHubba plugin, including hypoxia-inducible factor 1 subunit alpha (HIF1A), estrogen receptor 1 (ESR1), forkhead box O1 (FOXO1), insulin-like growth factor 1 (IGF1), CREB binding protein (CREBBP), SMAD family member 2 (SMAD2), Kruppel like factor 4 (KLF4), ATM serine/threonine kinase (ATM), platelet-derived growth factor receptor beta (PDGFRB), protein tyrosine kinase 2 (PTK2) (**Figure 6B** and **Supplementary Table 5**). Finally, we obtained the hsa_circRNA_0007990-microRNA-mRNA network using the Cytoscape 3.9.1 software, including five microRNAs and 93 mRNAs containing ten key circRNA-microRNA-mRNA regulatory axes (**Figure 6C**). The top ten hub genes in the hsa_circRNA_0007990 ceRNA network were associated with inflammation by searching the previous studies (**Supplementary Table 6**).

## Discussion

Our study showed 412 differentially expressed circRNAs in the peripheral blood between UIA patients and healthy controls by circRNA microarray. Twelve differentially expressed circRNAs were associated with AWE on HR-VMI in UIA patients. Hsa_circ_0007990 had the most remarkable fold change among the differentially expressed exonic circRNAs. The expression of hsa_circ_0007990 was increased gradually in the healthy control, UIA without AWE, and UIA with AWE. We predicted Ago2, DGCR8, EIF4A3, and PTB as the RNA binding proteins and period circadian regulator 1 as an encoding protein with hsa_circ_0007990. The hsa_circ_0007990-microRNA-mRNA network containing five microRNAs (miR-4717-5p, miR-1275, miR-150-3p, miR-18a-5p, miR-18b-5p), and 97 mRNAs was constructed. The ten hub genes, including HIF1A, ESR1, FOXO1, IGF1, CREBBP, SMAD2, KLF4, ATM, PDGFRB, and PTK2, were all associated with the inflammatory response. These results suggest that hsa_circ_0007990 may be a novel inflammatory blood biomarker for assessing UIA patients.

AWE on HR-VWI has been used as an imaging marker for vessel wall inflammation and aneurysm instability in patients with UIA. Recent studies showed the association of inflammatory blood biomarkers with AWE on HR-VWI in UIA patients (9–13). The plasma concentration of anti-inflammatory cytokine interleukin (IL)-10 in the aneurysm sac decreased in UIA with AWE (10). Our previous study showed that the neutrophil-lymphocyte ratio as an inflammatory peripheral blood biomarker was associated with AWE on HR-VWI in UIA patients (12). The up-regulated proinflammatory IL-1β and down-regulated anti-inflammatory IL-1RA in the serum were associated with AWE on HR-VWI in 34 patients with UIA. There was histological inflammation infiltration in aneurysm tissues with AWE. The serum IL-1β, IL-1RA, and IL-1RA/IL-1β ratio were associated with the inflammation-related proteins in aneurysm tissues (13). These results suggested that systematic inflammatory cytokines in the peripheral blood correlated with inflammatory factors in aneurysm tissues with AWE on HR-VWI, which may be a potential biomarker for aneurysm instability in UIA. In this study, we assessed the relationship of blood circRNAs as an inflammatory biomarker with AWE on HR-VWI in UIA patients.

CircRNAs play a key role in forming and rupturing IA. CircRNAs modulated phenotype, proliferation, migration, invasion, and apoptosis of vascular smooth muscle cells in aneurysm tissues (14–16). Inflammation is a vital pathological mechanism in IA rupture. Differentially expressed circRNAs related to IA have been shown to involve the inflammatory pathways and regulate the inflammatory gene expression through the circRNA-microRNA-mRNA axis (17–19). CircRNA_0079586 and circRNA_RanGAP1 in the endothelial cell of IA tissues were involved in the inflammatory response and IA rupture *via* miR-183-5p/myeloperoxidase and miR-877-3p/myeloperoxidase, respectively (17). CircLIFR in the IA tissues regulated the proliferation, migration, invasion, and apoptosis of vascular smooth muscle cells *via* the axis of miR-1299 and kinase insert domain receptor (18).

CircRNA hsa_circ_0007990 as a peripheral blood biomarker was associated with vessel wall inflammation on HR-VWI in this study. The ten hub genes in the predicted hsa_circ_0007990-microRNA-mRNA network were all involved in the inflammatory response (**Supplementary Table 6**). Recent studies suggested that circRNA may be used as a peripheral inflammatory blood biomarker for assessing IA instability. CircRNA expression profile of the peripheral blood in patients with multiple IA has been identified. The predicted dysregulated genes were mainly involved in the inflammatory response (21). Two hundred thirty-five differentially expressed circRNAs were shown in patients between ruptured IA and UIA by a circRNA microarray analysis of the peripheral blood. The mammalian target of rapamycin signaling pathway was one of the most significant pathways predicted by the targeted genes (22). Several differentially expressed circRNAs in IA tissues regulated inflammatory pathways. In the peripheral blood, two of these circRNAs (hsa_circ_0008433 and hsa_circ_0001946) were risk factors for IA rupture (19, 20).

In this study, the hsa_circ_0007990-microRNA-mRNA regulatory network consisted of five microRNAs and 97 mRNAs. CircRNAs play their functional effects *via* microRNA sponge activity to regulate gene transcription. Functional analysis indicated the top five predicted microRNAs (miR-18a-5p, miR-18b-5p, miR-150-3p, miR-1275, miR-4717-5p) binding on hsa_circRNA_0007990 through the CircBank database. MiR-18b-5p was shown as one of the significantly downregulated plasma microRNA biomarkers in ruptured IA, and its target genes were involved in inflammatory signaling pathways (26). Macrophage-derived miR-18a-5p was a potential urinary marker of early acute kidney injury following renal transplantation, and its several targets were involved in the cell cycle (27). MiR-150-3p was reported to involve in cyclophosphamide-induced lung inflammation and fibrosis *via* NF-κB and MAPK signaling pathways (28). In addition, five hub genes (HIF1A, ESR1, FOXO1, IGF1, and CREBBP) were identified in the hsa_circ_0007990-microRNA-mRNA regulatory network. HIF1A was upregulated in ruptured IAs and played an essential role in the PI3K-Akt signaling pathway (29, 30). FOXO1 increased in IA tissues and blocked myeloid cell leukemia sequence 1 transcription to involve the proliferation, apoptosis, and inflammation of vascular smooth muscle cells. MiR-124-5p downregulated FoxO1 expression to inhibit the migration and invasion of endothelial cells and inflammatory response (31, 32). IGF-1 alleviated the neuroinflammation and microglial activation induced by aneurysmal subarachnoid hemorrhage (33).

This study has some limitations. The sample size in this study was small with a single center data. The inflammatory response may promote intracranial aneurysm formation, progression, and rupture. In this study, the aneurysm size was significantly larger in patients with AWE than those without AWE (6.15 mm vs. 4.15 mm, *P*=0.025). Circ_0007990 expression was significantly higher in the UIA with AWE, which also tended to be associated with a larger size of the aneurysm. In one study, hsa_circ_0021001 expression in peripheral blood was not significantly associated with the size of the aneurysm (34). The relationship of differentially expressed circRNAs with a larger size of UIA needs to assess during aneurysm progression induced by inflammation. Three differentially expressed circRNAs (hsa_circ_0008433, hsa_circ_0001946, and hsa_circ_0021001) may be used as the diagnostic blood biomarkers for IA and the risk factors for aneurysms rupture (20, 34). The potential diagnostic value of 12 differentially expressed circRNAs identified from our study for IA diagnosis needs further assessment. The expression of predicted key microRNAs, hub mRNAs, and RNA binding proteins with hsa_circ_0007990 needs further confirmation in larger UIA samples. The exact mechanism of hsa_circ_0007990 involved in the inflammation pathway is still needed for further *in vitro* and *in vivo* study.

## Conclusions

Differentially expressed blood circRNAs associated with AWE on HR-VWI can be used as novel inflammatory biomarkers for assessing UIA patients. The mechanism of hsa_circRNA_0007990 for UIA progression needs to investigate further.

## Data availability statement

The datasets presented in this study can be found in online repositories. The names of the repository/repositories and accession number(s) can be found in the article/**Supplementary Material**.

## Ethics statement

The studies involving human participants were reviewed and approved by Sun Yat-sen Memorial Hospital, Sun Yat-sen University. The patients/participants provided their written informed consent to participate in this study.

## Author contributions

Z-SS participated in the design of the study. Z-SS, X-BW and Y-TW wrote the initial manuscript. Z-SS revised the manuscript. All authors critically reviewed and edited the manuscript, approved the final version, participated in the interpretation and collection of the data. All authors contributed to the article and approved the submitted version.

## Funding

Z-SS is supported by the National Natural Science Foundation of China (Grant Numbers: 81873752 and 81720108014) and the Science and Technology Planning Project of Guangzhou City (Grant Number: 201704020166).

## Conflict of interest

The authors declare that the research was conducted in the absence of any commercial or financial relationships that could be construed as a potential conflict of interest.

## Publisher’s note

All claims expressed in this article are solely those of the authors and do not necessarily represent those of their affiliated organizations, or those of the publisher, the editors and the reviewers. Any product that may be evaluated in this article, or claim that may be made by its manufacturer, is not guaranteed or endorsed by the publisher.
