# Supplementary material for: Circular RNA hsa_circ_0007990 as a blood biomarker for unruptured intracranial aneurysm with aneurysm wall enhancement
Source: Front Immunol. 2022 Nov 17;13:1061592. doi: 10.3389/fimmu.2022.1061592 (PMC9714537; doi:10.3389/fimmu.2022.1061592)
Supplement: Supplementary file 1 [file Table_1.docx]

**Supplementary Table 1** qRT-PCR primer sequences.

|  | Primer sequence | Product length |
| --- | --- | --- |
| has_circ_0007990 | Forward:5’GGTGATGCTCTGCCGCTA3’ | 108 |
|  | Reverse:5’CTCATACTTACAGTCGTCCCG3’ |  |
| GAPHD | Forward:5’GGGAAACTGTGGCGTGAT3’ | 299 |
|  | Reverse:5’GAGTGGGTGTCGCTGTTGA3’ |  |
